# Home Sweet Home: The Tumor Microenvironment as a Haven for Regulatory T Cells

**DOI:** 10.3389/fimmu.2013.00197

**Published:** 2013-07-16

**Authors:** Beatrice Ondondo, Emma Jones, Andrew Godkin, Awen Gallimore

**Affiliations:** ^1^Nuffield Department of Medicine, The Jenner Institute (ORCRB), University of Oxford, Oxford, UK; ^2^Infection and Immunity, School of Medicine, Henry Wellcome Building, Cardiff University, Cardiff, UK

**Keywords:** tumor immunology, regulatory T cells, intra-tumoral proliferation, chemokines, immunotherapy of cancer

## Abstract

CD4^+^Foxp3^+^ regulatory T cells (T_regs_) have a fundamental role in maintaining immune balance by preventing autoreactivity and immune-mediated pathology. However this role of T_regs_ extends to suppression of anti-tumor immune responses and remains a major obstacle in the development of anti-cancer vaccines and immunotherapies. This feature of T_reg_ activity is exacerbated by the discovery that T_reg_ frequencies are not only elevated in the blood of cancer patients, but are also significantly enriched within tumors in comparison to other sites. These observations have sparked off the quest to understand the processes through which T_regs_ become elevated in cancer-bearing hosts and to identify the specific mechanisms leading to their accumulation within the tumor microenvironment. This manuscript reviews the evidence for specific mechanisms of intra-tumoral T_reg_ enrichment and will discuss how this information may be utilized for the purpose of manipulating the balance of tumor-infiltrating T cells in favor of anti-tumor effector cells.

## What are T_regs_?

Regulatory T cells (T_regs_) are suppressor cells that are necessary for maintaining immune homeostasis and immunological tolerance to self and which play a key role in limiting excessive and harmful immune responses (1). Several different types of suppressor T cells have been described including cells within both CD4^+^ and CD8^+^ populations (2). The most prominent of these express both CD4 and Foxp3 and can arise either in the thymus or in the periphery (1). A recent correspondence in *Nature Immunology* recommended the adoption of new nomenclature for T_regs_ (3). The authors suggested that thymus-derived T_reg_ cells be called tT_reg_ (rather than nT_reg_) to denote those that are thymus-derived and pT_reg_ for those that differentiate in the periphery [therefore replacing the terms i(induced)T_reg_ and a(adaptive)T_reg_]. This review will, as far as possible, adopt the recently recommended nomenclature. In addition the term T_reg_ will only be used to describe cells where suppressor activity has been demonstrated either *in vivo* or *in vitro* and where suppressor function has not been confirmed, the cells will be termed Foxp3^+^CD4^+^ T cells.

## Promotion of Tumor Progression by T_regs_

There is an emerging consensus that effective anti-tumor immunity is characterized by a T_helper_1 (Th1)/CD8^+^ T cell response (4). This type of response however, is susceptible to suppression by T_regs_ and several studies using mouse models have shown that partial or complete removal of this inhibitory influence uncovers anti-tumor immune responses capable of preventing tumor development and limiting tumor progression (5–7). Approaches aimed at modulating Foxp3^+^ T cell frequencies in patients with cancer have been shown to enhance vaccine-induced anti-tumor immune responses and even boost endogenous responses (8–11). These exciting findings underpin the importance of fully understanding the role of T_regs_ in cancer so that these cells can be manipulated in order to optimize cancer immunotherapy.

## Mechanisms of Foxp3^+^ T Cell Enrichment within Tumors

Studies have shown that progressing mouse and human tumors can be associated with enhanced T_regs_ activity and escalating immune suppression (12, 13). Indeed Foxp3^+^ T cells manage to successfully pervade, and often dominate the tumor-specific immune response; Foxp3^+^ to Foxp3^−^ T cell ratios in the range 0.5–1:1 have been described in some tumors (12, 14, 15). A few theories have been proposed to explain how Foxp3^+^ T cells become enriched in tumors and in the peripheral blood of tumor-bearing hosts. There may be preferential migration of Foxp3^+^ T cells to tumors in response to chemokines expressed by tumor cells and stroma. Foxp3^+^ T cells, preferentially attracted to the tumor microenvironment may use the same or additional cues to aid their retention within the tumor mass. In addition, tumor establishment may trigger production of a cocktail of factors that support increased Foxp3^+^ T cell proliferation and/or the conversion of conventional Foxp3^−^CD4^+^ T cells into Foxp3^+^ cells. Various lines of supporting evidence exist for these mechanisms of Foxp3^+^ T cell enrichment in tumors and will be discussed in this review.

## Chemokine-Mediated Recruitment of Foxp3^+^ T Cells into Tumors

Migration of cells into peripheral tissues and sites of inflammation depends on their expression of various chemokine receptors, selectins (and selectin ligands), and integrins. Generally, effector-like inflammation-seeking T cells (including T_regs_) express inflammatory chemokine receptors and adhesion molecules that enhance their capacity to migrate to inflamed tissues (16–24). Different tumors are characterized by unique albeit overlapping chemokine signatures. Tumor cells and surrounding stromal cells can express these chemokines, which serve to facilitate migration and accumulation of various leukocytes in the tumor (25–27). While some of these leukocytic infiltrates comprise macrophages (28) and myeloid derived suppressor cells (MDSCs) (29) which promote tumor progression and metastasis, a high frequency of infiltrating CD3^+^ T cells often correlates with improved clinical outcome, e.g., in ovarian and colorectal cancer (CRC) (30, 31). Whether or not the degree of CD3^+^ T cell infiltrate correlates with strong anti-tumor immunity may also depend on the frequency and suppressive capacity of tumor-infiltrating T_regs_. Consequently, increased infiltration of Foxp3^+^ T cells is often associated with a poor prognosis and accelerated tumor progression (32).

## Inflammatory Chemokines and Their Receptors

### CCR4

CCR4 has been shown to be expressed on a greater proportion of T_regs_ than conventional T cells and to be important for guiding T_regs_ to sites of inflammation (24, 33). Several studies indicate that the tumor-expressed chemokines CCL22 and CCL17, which are ligands for CCR4, play a role in the recruitment and enrichment of T_regs_. A study by Curiel and colleagues, clearly demonstrated a major role for CCL22 in recruitment of CCR4^+^ T_regs_ into human ovarian carcinomas (13). CCL22 alone, or in combination with CCL17, has been implicated in T_reg_ recruitment to human breast (34, 35) and prostate (36) cancers. Increased levels of CCL17 and/or CCL22 are also associated with higher frequencies of CD4^+^Foxp3^+^ T cells in cerebral spinal fluid of patients with lymphomatous and carcinomatous meningitis (37), gastric (38), and esophageal squamous cell carcinomas (39). Using mouse models, several approaches, including the use of specific antibodies, antagonists, or siRNA, have been used to block the CCL22/CCL17 – CCR4 axis, resulting in reduction in T_reg_ frequencies and a concomitant increase in anti-tumor activity (40–42).

### CCR5

Disruption of CCR5/CCL5 signaling has also been shown in mouse models to impair intra-tumoral T_reg_ accumulation and slow tumor progression (43). Similarly, CCL5 levels correlate with increased T_reg_ frequencies and impaired CD8^+^ T cell responses in human colon cancer (44). Further evidence for CCR5-dependent T_reg_ enrichment comes from a study exploring the potential mechanisms through which MDSCs inhibit anti-tumor immunity. MDSCs infiltrating mouse RMA-S lymphomas were shown to increase the levels of CCL3, CCL4, and CCL5, which in turn enhanced the recruitment of CD4^+^Foxp3^+^ T cells via CCR5 (45). CCR5 deficiency (demonstrated by use of CCR5^−/−^ mice) or CCL5 blockade (using Met-RANTES) led to diminished CD4^+^Foxp3^+^ T cells numbers and slower tumor growth (45). However, apart from its ability to attract CD4^+^Foxp3^+^ T cells to tumors, the pivotal role for CCR5 in mediating recruitment and activation of conventional T cells dictates that CCR5 is also important for achieving strong anti-tumor immune responses and regression of established tumors (46–49). Thus, although the findings of some mouse models indicate that the CCR5 axis can be targeted to reduce T_reg_ accumulation, the general utility of this approach is likely to be limited by the potential for concurrent effects on anti-tumor effector cells.

### CXCR3

A similar situation applies to the chemokine receptor CXCR3. Intra-tumoral accumulation of CXCR3^+^Foxp3^+^ T cells has been reported in human ovarian, colorectal, and hepatocellular carcinomas (50, 51). However, like CCR5, CXCR3 is abundantly expressed on activated cells, binding the IFN-γ-induced chemokines, CXCL9, CXCL10, and CXCL11. Indeed, homing and migration of activated effector cells (CTL, NK, NKT, and T helper) is highly dependent on CXCL9/CXCL10/CXCL11 – CXCR3 signaling thereby limiting the utility of this pathway for targeted prevention of T_reg_ recruitment. CXCR3 and CCR5 are often co-expressed by effector T cells. In a study of human colorectal carcinomas expressing CXCL10 and CCL5, the CD8^+^IFNγ^+^ T cell infiltrate comprised predominantly CXCR3^+^CCR5^+^ cells (52), concurrent with a favorable prognosis as previously described (30). Similarly, CXCL9, CXCL10, and CXCL11 expression by sporadic human renal cell carcinomas was associated with increased frequency of CXCR3^+^CCR5^+^ T cells and a favorable prognosis which was characterized by the absence of recurrences following curative surgery (53). Furthermore tumor-expressed CXCL9 was shown to be crucial for immune control of murine cutaneous fibrosarcomas (54) while CXCL11 secretion by genetically modified mouse T cell lymphoma cells (EL4) led to increased infiltration of CD8^+^CXCR3^+^ T cells and subsequent tumor rejection (55). Considering the body of evidence highlighting a favorable prognosis for cancers expressing these IFN-γ induced chemokines, disruption of the CXCR3 and/or CCR5 pathways to prevent T_reg_ accumulation in tumors is unlikely to be effective for promoting tumor immunity.

## Hypoxia-Induced Chemokines and Their Receptors

### CCR10

Hypoxia and angiogenesis are both characteristic features of advanced solid tumors. Both of these features also serve to modulate the enrichment of intra-tumoral T_regs_ expressing CCR10. CCL28, a chemokine known to be upregulated by hypoxia has recently been shown to recruit CCR10^+^ T_regs_ to mouse ovarian cancers (56). These CCR10^+^ T_regs_ contributed to tumor progression by secreting vascular endothelial growth factor A (VEGF-A), thereby promoting angiogenesis. Understanding the nature of the relationship between VEGF-A and T_regs_ may prove important. VEGF-A blockade not only reduced angiogenesis but also has been shown to reduce the extent of T_reg_ infiltration in mouse models resulting in enhanced vaccine-induced immune responses (57). Moreover, treatment of CRC patients with the anti-VEGF-A monoclonal antibody bevacizumab reversed T_reg_ accumulation in patients’ blood (58) whilst VEGFR2^+^CD4^+^Foxp3^+^ cells are reportedly associated with poor prognosis in CRC (59) supporting the theory that angiogenic factors may be targeted for the purpose of modulating both angiogenesis and the anti-tumor immune response.

### CXCR4

Vascular endothelial growth factor A has also been shown to work synergistically with CXCL12, a chemokine commonly expressed by tumors, to promote tumor angiogenesis (60). In a study of patients with basal-like breast cancers, infiltration with Foxp3^+^ cells was shown, as above, to correlate with tumor hypoxia (61). In this study however, a preferential accumulation of Foxp3^+^ cells expressing CXCR4, the receptor for CXCL12, was observed. The authors further showed that accumulation of these CXCR4^+^Foxp3^+^ cells was associated with a poor prognosis. Although CD8^+^ and Foxp3^−^CD4^+^ T cells can also express CXCR4, there are reports that CXCL12 preferentially attracts T_regs_ to human lung adenocarcinomas (62) and advanced cervical cancers (63), thereby indicating that targeting the CXCR4–CXCL12 axis may represent a useful means of selectively reducing the intra-tumoral T_reg_ infiltrate. In support of this, using a mouse model of ovarian cancer, Righi and colleagues showed that administration of a specific CXCR4 antagonist, AMD3100 (64), was associated with several anti-tumor effects including increased tumor cell death, reduced dissemination and angiogenesis and better survival of the treated animals (65). Significantly, the authors also observed a selective reduction in the recruitment of Foxp3^+^ T cells in comparison with CD8^+^ T cells (65).

The picture that emerges from these reports is that tumors with high levels of T_regs_, recruited in response to hypoxia (via CCR10 and/or CXCR4), are rich in VEGF-A and therefore, serve to drive neovascularization. Such a pathway implies that angiogenesis and the recruitment and activity of T_regs_ work side-by-side, facilitating tumor growth directly through neovascularization and indirectly through promoting immune suppression. With this in mind, it may prove useful to further explore potential synergy between therapies targeting angiogenesis and those targeting T_regs_.

## Lymphoid-Associated Chemokines and Their Receptors

### CCR7

The role of CCL21/CCR7 signaling in the recruitment and accumulation of T_regs_ in tumors has been described in one study using B16 melanomas engineered to express higher levels of CCL21. These tumors recruited high numbers of T_regs_ and progressed more rapidly compared to tumors expressing normal or lower CCL21 levels (66). In contrast, other studies indicate that the CCL21/CCR7 pathway promotes increased tumor control as a result of increased recruitment of effector immune cells (67). Furthermore, intra-tumoral expression of CCL21 boosted CTL responses after DNA vaccination of mice and induced regression of B16 melanomas (68). In another study, intra-tumoral delivery of CCL21 inhibited lung cancer growth in mice. Inhibition of tumor growth was associated with reduced frequencies of T_regs_ and MDSC but enhanced recruitment of CCR7^+^Foxp3^−^ T cells (69). Moreover, a recent study of patients with metastatic CRC indicated that tumor infiltration with CCR7^+^ T cells was associated with a more favorable prognosis (70). Given the plethora of studies highlighting the important role of CCL21 in recruitment of immune effector cells and subsequent tumor immunity and the paucity of studies to support enhanced T_reg_ recruitment to the tumor via CCL21/CCR7, it is highly unlikely that selective targeting of this pathway as a means to prevent T_reg_ recruitment will be of clinical benefit in cancer patients.

Whether chemokines lead to the preferential enrichment of T_regs_ in tumors is as yet unclear although there is evidence that T_reg_ recruitment to tumors may be selectively inhibited through chemokine receptor blockade: the most notable candidates being CCR4, CXCR4, and CCR10. Such strategies may not however, impinge on the existing pool of tumor-infiltrating T_regs_. Chemokines may perform functions other than to attract T_regs_ to tumors. It is highly likely for example, that chemokines, expressed intra-tumorally, serve to retain T_reg_ cells, perhaps preferentially, within the tumor mass. If this is the case, selective retention of T_regs_ in the tumor microenvironment could significantly influence their fate compared to that of conventional T cells, with clear immunosuppressive consequences. Although the key role of chemokines is to act as chemoattractants, a role for CCL5 in promoting T cell activation has been demonstrated; in these studies CCL5 was shown to induce signaling events in T cells in antigen-independent fashion (71, 72). This finding raises the intriguing possibility that chemokines present within the tumor microenvironment may influence T cell activity, including the activity of T_regs_.

## Induction of T_regs_ in the Tumor Microenvironment

The possibility that conversion of conventional T cells into T_regs_ represents a mechanism of T_reg_ enrichment in tumors has been explored. In adoptive transfer experiments, purified CD4^+^CD25^−^ T cells transferred into tumor bearing mice have been shown to convert into Foxp3^+^CD4^+^CD25^+^ cells within the tumor microenvironment (73, 74). In studies of patients with melanoma, Fourcade and colleagues demonstrated that CD4^+^CD25^−^ T cells and Foxp3^+^CD4^+^ T cells could recognize the same peptide and moreover, clonotypic analyses of these cells revealed a common T cell receptor (TCR) Vβ usage (75). These findings are compatible with the hypothesis that conventional tumor-specific T cells can convert into T_regs_. Whilst the potential for conversion of conventional T cells into T_regs_ is undoubtedly demonstrated in these types of studies, the extent to which this contributes to what is a significant intra-tumoral enrichment of Foxp3^+^ T cells is unclear. Addressing this question directly has been hampered by reports that Foxp3 can be transiently upregulated on activated T cells without necessarily conferring suppressor functions and a lack of definitive markers to discriminate tT_regs_ from pT_regs_. The “best” markers are the transcription factor, Helios, and the type 1 transmembrane protein, neuropilin 1 (Nrp1), which, according to some reports, are expressed mainly by tT_regs_ (76, 77). In the case of renal cell cancer patients, the significant increase in Foxp3^+^ T cells observed in both untreated and IL-2-treated patients are helios^+^ suggesting that tumors drive expansion of tT_reg_ and not pT_regs_ (78). The same observation has been made in studies of patients with glioblastoma multiforme (GBM) and parallel studies of orthotopic mouse models of brain tumors (79). Studies of Nrp1 expression have resulted in mixed findings where in some mouse tumors Nrp1^+^Foxp3^+^ T cells predominate whereas in others they do not (80, 81). The validity of both helios and Nrp1 as true discriminators of tT_regs_ versus pT_regs_ has however been disputed, thus no definitive conclusions can be drawn from the studies described above (82, 83).

Working on the premise that pT_regs_ and conventional T cells share the same TCRs, we used a mouse model of carcinogen-induced tumors to compare the TCR repertoires of tumor-infiltrating Foxp3^−^ and Foxp3^+^CD4^+^ T cells in order to determine the extent of TCR overlap between the two populations following their recovery from the tumor microenvironment (15). The data clearly indicated that the TCR repertoires of tumor-infiltrating Foxp3^−^ and Foxp3^+^CD4^+^ T cells are distinct, implying that at least in the case of carcinogen-induced tumors, conversion of conventional T cells is not a significant cause of intra-tumoral T_reg_ enrichment. This finding was confirmed in a similar analysis of CD4^+^Foxp3^+^ T cells recovered from TRAMP mice in which prostate cancer is driven by transgenic expression of SV40 large T antigen (84). In this study, thymic development of the CD4^+^Foxp3^+^ T cells was Aire-dependent and the cells appeared to be specific for a prostate-associated self-antigen. Overall, the results of this study support enrichment of intra-tumoral tT_regs_ as the main mechanism of T_reg_ accumulation in tumors rather than conversion of conventional T cells to pT_regs_.

Collectively, evidence to support conversion as a major mechanism of T_reg_ enrichment in tumors is currently weak. Most of the direct evidence for a limited role for conversion has however, come from mouse models. There are reports that human CD4^+^Foxp3^−^ cells can convert into CD4^+^Foxp3^+/lo^ T_regs_ *in vitro* and that the phenotypic characteristics of these cells can resemble CD4^+^ T cell sub-populations isolated from tumors (85). Whilst these data do not provide definitive answers relating to the relationship between different tumor-infiltrating T cell subpopulation (such as that gained from TCR clonotyping), it remains possible that in human cancers, there is some enrichment of pT_regs_.

## Superior Proliferation of tT_regs_ within the Tumor Microenvironment

Given the evidence that Foxp3^+^CD4^+^ T cells gain an edge in accessing the tumor microenvironment through a combination of differential chemokine receptor expression and an increased capacity to migrate in response to hypoxia-induced chemokines and VEGF-A, it is reasonable to speculate that migration does contribute to their observed enrichment within the tumor microenvironment. Higher frequencies of Foxp3^+^CD4^+^ T cells are however also observed in spleen/blood and draining lymph of tumor-bearing mice and patients with cancer compared to non-tumor-bearing controls. In carcinogen-induced tumors, enhanced proliferation of CD4^+^CD25^+^ T cells has been reported (86). Studies examining proliferation of intra-tumoral Foxp3^+^ T cells in brain tumors imply that the majority of proliferating cells are helios^+^; for example in mouse models of glioblastoma, it has been reported that the majority of tumor-infiltrating Foxp3^+^ T cells express helios and are highly proliferative, significantly more so than helios^−^Foxp3^+^ and Foxp3^−^CD4^+^ T cells (79). Moreover, if these highly proliferative CD4^+^Foxp3^+^helios^+^ cells are, as this study suggests, suppressive within the tumor microenvironment then the available evidence favors intra-tumoral expansion of tT_reg_ as a major mechanism of T_reg_ enrichment in tumors.

Why might CD4^+^Foxp3^+^ T_regs_ demonstrate enhanced proliferation in the tumor microenvironment compared to CD4^+^Foxp3^−^ T cells? Evidence suggests that the T_reg_ population contains a higher number of cells that respond to self-antigens compared to Tconv cells (87, 88). Thus, in the case of tumors, T_regs_ may receive stronger antigen-driven signals than conventional T cells, promoting their expansion in tumors. Using a mouse model of melanoma (B16), Ghiringhelli and colleagues showed that tumors can license dendritic cells (DCs) to promote the proliferation of T_regs_ through the production of TGF-β (89). Another study, also utilizing the B16 tumor cell line showed that plasmacytoid DCs promoted T_reg_ activation in an indoleamine 2,3-dioxygenase (IDO)-dependent manner (90). Whilst both studies assessed T_reg_ activity in tumor-draining lymph nodes, it is possible similar signals serve to further promote T_reg_ cell proliferation and survival within the tumor microenvironment. Of note, IDO production by human monocyte-derived DCs has also been shown to drive proliferation of highly suppressive CD4^+^Foxp3^+^ T cells (91). In addition, to these pathways, it has recently been shown that VEGFR^+^ T_regs_, purified from tumor-bearing mice proliferated in response to VEGF. The same study also demonstrated reduced T_reg_ frequencies in the peripheral blood of CRC patients treated with the VEGF-A blocking antibody, bevacuzimab (58).

Any signal that serves to promote T_reg_ activity, also therefore serves to indirectly suppress the activities of conventional T cells, one effect of which is to reduce local production of IL-2. Through expression of high levels of CD25, T_regs_ may out-compete conventional T cells for the limited supply of IL-2. Thus, within the tumor microenvironment T_regs_ may gain superiority by utilizing the available IL-2 to support their own proliferation and moreover, to further promote their immunosuppressive capability (92).

## Foxp3^+^ T_regs_ – The Tip of the Iceberg

**Figure 1:**
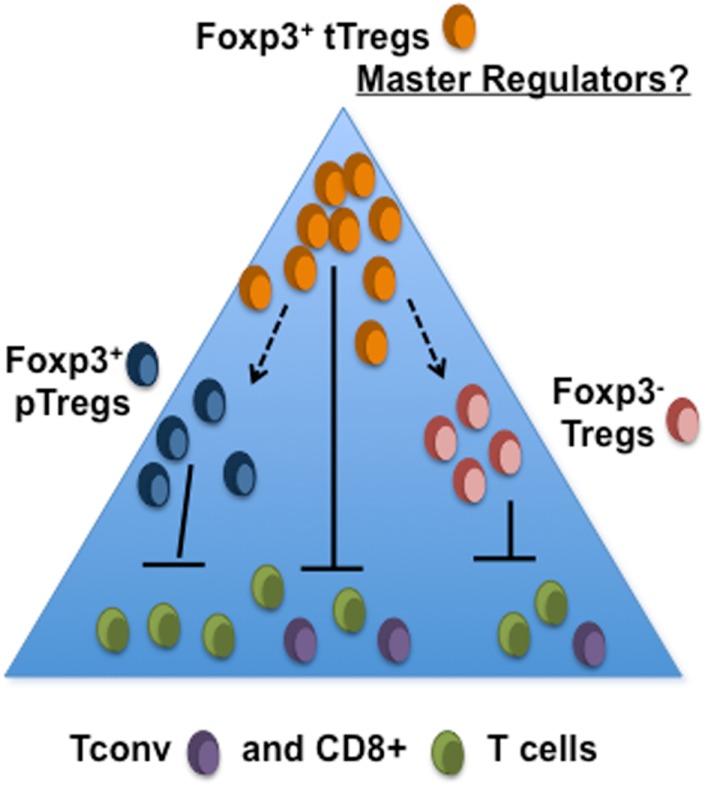
**Are intra-tumoral Foxp3^+^ t_regs_ simply the tip of the iceberg?** The tumor microenvironment may consist of several subsets of T_reg_ that serve to suppress the activities of tumor-specific CD4^+^ and CD8^+^ T cells. It is not yet known whether development of pT_reg_ or suppressor activity within CD4^+^Foxp3^−^ T population requires the presence of tT_reg_.

Although most available evidence indicates that the bulk of tumor-infiltrating Foxp3^+^ T_regs_ are tT_reg_, this does not preclude a significant immunosuppressive role for pT_reg_ or indeed Foxp3^−^ cells. With the possible exception of melanoma, there is a distinct paucity of publications detailing the phenotypic and functional characteristics of tumor-infiltrating T cells, particularly tumor-infiltrating CD4^+^ T cells. There are however suggestions that Foxp3^+^ T_regs_, whether pT_regs_ or tT_regs_, are not the only suppressive CD4^+^ T cell sub-population found in tumors. Using a transgenic mouse model of prostate cancer, Donkor et al. showed that TGF-β-blockade in Foxp3^−^ T cells resulted in heightened CTL responses and better immune-mediated control of primary and metastatic tumors (93). In human studies, elevated frequencies of CD4^+^ T cells expressing latency associated peptide (LAP) have been observed in blood of CRC patients compared to healthy controls. Interestingly, many of these did not express Foxp3 but could suppress proliferation of LAP^−^ cells in a TGF-β-dependent fashion (94). Moreover the LAP^+^CD4^+^ sub-population cells were also found in colorectal tumors where their proportions within the CD4^+^ tumor-infiltrating T cell pool increased with disease progression (94). Similarly, in a study of patients with hepatocellular carcinoma, elevated frequencies of intrahepatic CD4^+^Foxp3^−^ cells were observed in cancer patients compared to hepatitis C virus infected individuals with chronic liver disease; these CD4^+^Foxp3^−^ T cells expressed IL-10 and were suppressive *in vitro* (95). Collectively the data thus far, support a major role for the immunosuppressive cytokines IL-10 and TGFβ in mediating the suppressive effects of CD4^+^Foxp3^−^ T cells. Whether Foxp3^−^ suppressor T cells arise through sustained but inadequate activation of conventional T cells and/or through mechanisms of infectious tolerance is unknown. It is extremely important however, to determine whether or not Foxp3^+^ T_regs_ are responsible, directly or indirectly, for driving the acquisition of suppressor functions of tumor-infiltrating Foxp3^−^CD4^+^ T cells. This information will reveal whether or not modulating Foxp3^+^ T_regs_ will be sufficient for overcoming the influence of intra-tumoral suppressor T cells or whether multiple suppressor T cell subsets will need to be independently targeted (Figure 1).

## Implications for Cancer Immunotherapy

Strategies aimed at non-specifically targeting pathways of tolerance induction have proven extremely informative and potentially useful methods of cancer immunotherapy. Boosting conventional T cell activity through use of CTLA4-blocking antibodies can be highly effective in the treatment of metastatic melanoma (96, 97). Similarly, early findings with PD-1- or PDL1-blockade has shown clinical efficacy in melanoma patients without the toxicities observed with anti-CTLA4 antibody treatment (98). These differential toxic effects of CTLA4- versus PD-1-blockade reflect the phenotypes described for CTLA4- and PD-1-deficient mice. Whereas mice lacking CTLA4 exhibit systemic T cell proliferation (99, 100), those lacking PD-1 exhibit milder symptoms (101). This difference may be due to the ability of CTLA4 blockade to induce global T cell activation whereas PD-1 blockade serves to promote effector T cell responses. Differential effects of CTLA4- and PD-1-blockade on T_regs_ are also likely to contribute.

**Figure 2:**
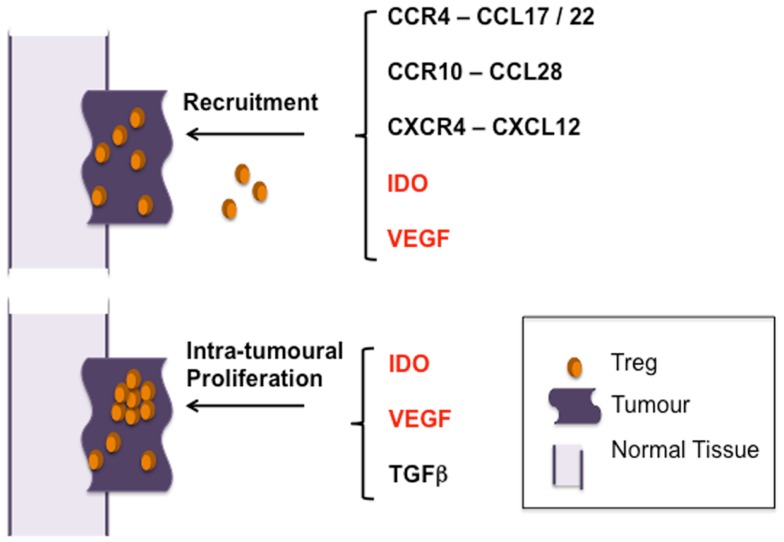
**Pathways of T_reg_ enrichment in tumors**. Studies thus far indicate that selective migration of T_reg_ and preferential proliferation of t_Treg_ result in their accumulation in tumors – the main pathways identified thus far are indicated. Mechanisms promoting both recruitment and proliferation are indicated in red.

Has our understanding thus far of intra-tumoral Foxp3^+^ T cell-enrichment identified mechanisms through which their potential influence on the anti-tumor immune response can be modulated and used to improve current T cell-orientated treatments (Figure 2)? Blockade of recruitment may be possible; administration of methyl gallate has been shown to inhibit recruitment of CD4^+^Foxp3^+^ cells through modulating expression of CCR4 whilst AMD3100 can antagonize the CXCR4–CXCL12 interaction (41, 65). VEGF-A blockade may also reduce the numbers of tumor-infiltrating T_regs_ through effects on both recruitment (57, 80) and proliferation (58). Moreover, this approach may also serve to enhance homing of anti-tumor T cells, possibly due the effects of its blockade on normalization of tumor blood vessels (102, 103). It may also be the case that targeting blood vessels can alter the composition of the intra-tumoral T cell pool. Recently we found that carcinogen-induced tumors were controlled in a proportion of mice in which T_regs_ had been largely ablated. The tumors of these mice, unlike progressing tumors, were distinguished by the presence of high endothelial venules (HEV); specialized blood vessels normally found only in lymph nodes that when present in tumors facilitated entry of anti-tumor effector cells (5). Thus, disabling T_regs_ can, directly or indirectly, impact on blood vessel differentiation, promoting access of anti-tumor T cells.

As well as VEGF-A, IDO has been implicated in both the recruitment and activation of T_regs_, underpinning the potential for targeting these molecules for modulating T_reg_ numbers within tumors (57, 80, 104) (Figure 2). Thus, inhibition of IDO, shown to be successful in promoting tumor-immunity in many mouse models, may prove a useful therapeutic target (105).

The enhanced proliferative activity of CD4^+^Foxp3^+^ T cells can also be exploited as a means of targeting T_regs_ with chemotherapeutic drugs such as cyclophosphamide. The findings of a recent study suggest that modulating T_reg_ numbers in this way can be successfully combined with vaccination strategies aimed at inducing or boosting anti-tumor effector T cells (11). It was demonstrated, in a phase 2 trial involving patients with renal cell carcinoma, that a single dose of cyclophosphamide reduced T_reg_ numbers and promoted immune responses to a peptide-based vaccine. These immune responses were associated with longer overall survival (11). Collectively, the data described herein point to the importance of exploring immunotherapeutic strategies aimed at modulating T_reg_ numbers, boosting anti-tumor T cell responses through vaccination and influencing blood vessel differentiation for the purpose of facilitating access of effective anti-tumor T cells to the tumor microenvironment.

As discussed above, it appears that in terms of T cell-mediated immunosuppression in the tumor microenvironment, Foxp3^+^ T_regs_ are just one subpopulation of suppressor T cell. It is likely that the tumor-infiltrating CD4^+^ T cell pool is highly heterogeneous comprising both Foxp3^+^ and Foxp3^−^ suppressor cells. It is not surprising therefore that even in mouse models whereby Foxp3^+^ T_reg_ cells can be specifically and almost completely ablated that effects on tumor growth are often modest and in the majority of cases, despite the systemic autoreactivity induced by Foxp3^+^ T_reg_ depletion, tumors continue to grow (5– 7). It is important therefore to determine whether ablation of Foxp3^+^ T cells also reduces or removes the immunosuppressive influence of Foxp3^−^ T_regs_. Moreover, the nature of anti-tumor T cell responses is not completely understood. Whilst it is clear that Th1/CD8^+^ T cell responses can exert potent anti-tumor activities, some reports also suggest that Th17 cells can also participate in limiting tumor progression (106, 107). As reported recently in a study of patients with pancreatic cancer, Th17 cells may also represent relevant targets for suppression by T_regs_ (108). With these questions in mind, it is imperative that we continue to characterize tumor-infiltrating T cell pools with respect to deciphering the origins, specificities, and phenotypes of both Foxp3^+^ and Foxp3^−^ T_regs_ cells and their targets. Such studies may reveal new means of disabling intra-tumoral T_regs_.

Overall, our current knowledge of T_regs_ indicates that there is room for optimism. Preclinical and clinical studies will continue to use current and new findings to examine both benefits and toxicities of combination therapies (e.g., immune modulation, blood vessel normalization, vaccination) aimed at redressing the balance between tolerance and immunity within the tumor microenvironment. Modulating T_reg_ numbers and activity is likely to represent an integral part of this process.

## Conflict of Interest Statement

The authors declare that the research was conducted in the absence of any commercial or financial relationships that could be construed as a potential conflict of interest.
